# Therapeutic Effect of Caffeine Treatment Immediately Following Neonatal Hypoxic-Ischemic Injury on Spatial Memory in Male Rats

**DOI:** 10.3390/brainsci3010177

**Published:** 2013-03-05

**Authors:** Michelle Alexander, Amanda L. Smith, Ted S. Rosenkrantz, R. Holly Fitch

**Affiliations:** 1 Department of Psychology, University of Connecticut, Storrs, CT 06269, USA; E-Mails: amanda.l.smith@uconn.edu (A.L.S.); roslyn.h.fitch@uconn.edu (R.H.F.); 2 Department of Pediatrics, University of Connecticut Health Center, Farmington, CT 06030, USA; E-Mail: rosenkrant@nso1.uchc.edu

**Keywords:** neuroprotection, hypoxic ischemic encephalopathy, Morris water maze, neonatal

## Abstract

Hypoxia Ischemia (HI) refers to the disruption of blood and/or oxygen delivery to the brain. Term infants suffering perinatal complications that result in decreased blood flow and/or oxygen delivery to the brain are at risk for HI. Among a variety of developmental delays in this population, HI injured infants demonstrate subsequent memory deficits. The Rice-Vannucci rodent HI model can be used to explore behavioral deficits following early HI events, as well as possible therapeutic agents to help reduce deleterious outcomes. Caffeine is an adenosine receptor antagonist that has recently shown promising results as a therapeutic agent following HI injury. The current study sought to investigate the therapeutic benefit of caffeine following early HI injury in male rats. On post-natal day (P) 7, HI injury was induced (cauterization of the right common carotid artery, followed by two hours of 8% oxygen). Male sham animals received only a midline incision with no manipulation of the artery followed by room air exposure for two hours. Subsets of HI and sham animals then received either an intraperitoneal (i.p.) injection of caffeine (10 mg/kg), or vehicle (sterile saline) immediately following hypoxia. All animals later underwent testing on the Morris Water Maze (MWM) from P90 to P95. Results show that HI injured animals (with no caffeine treatment) displayed significant deficits on the MWM task relative to shams. These deficits were attenuated by caffeine treatment when given immediately following the induction of HI. We also found a reduction in right cortical volume (ipsilateral to injury) in HI saline animals as compared to shams, while right cortical volume in the HI caffeine treated animals was intermediate. These findings suggest that caffeine is a potential therapeutic agent that could be used in HI injured infants to reduce brain injury and preserve subsequent cognitive function.

## 1. Introduction

Children born prematurely (less than 37 weeks gestational age) or at very low birth weight (VLBW; less than 1500 g) are at increased risk for hypoxic-ischemic (HI) injury, resulting from decreased blood and/or oxygen flow to brain tissue [1]. HI injury and encephalopathy (HIE) can also occur in full-term infants following birth complications (*i.e.*, asphyxia, cord prolapse or placental disruption) [2,3,4]. Cognitive delays are seen later in life in both premature and full-term HI injured populations. For example, children born prematurely showed reduced memory quotient scores on the Weshler’s Memory Scale in childhood [5], as well as deficits on a spatial working memory task [6]. Children born very preterm also showed later deficits on a visuospatial memory task when compared to healthy age-matched controls. Furthermore, the severity of their brain injury at birth negatively correlated with later test scores on verbal and visuospatial tasks [7]. Neural structures associated with memory abilities are also known to be affected by HI injury. For example, hippocampal volume was positively correlated with visual working memory scores in two year old children born prematurely (*i.e.*, smaller volumes/lower scores). Preterm infants with smaller hippocampal volumes also showed deficits on a visual working memory task when compared to non-injured infants [8]. Similar effects have been seen in full-term HI injured infants where severe injury (diagnosed at birth) was associated with later deficits in various memory tasks such as memory for names, narrative sentence repetition, and “everyday memory tasks”, when compared to scores from children with a mild-moderate injury [9]. 

Rodent models can be used to further investigate the effects of neonatal HI brain injury on subsequent behavioral and cognitive outcomes. One well characterized technique involves cauterization of the right common carotid artery on postnatal day (P) 7 rat (roughly corresponding 34–36 weeks gestational age in human infants), followed by a period of decreased oxygen exposure. This P7 HI model simulates both behavioral and pathophysiological outcomes as seen in term HI injured infants, including memory deficits [10,11]. For example, P7 HI animals performed worse on the Morris Water Maze (MWM; a test of spatial navigation and learning) compared to sham animals [12,13,14,15,16,17]. Along with spatial memory function, P7 HI injured animals also show deficits in choice reaction time tasks [17], place learning tasks [15], and spatial working memory tasks [15,16,17]. In addition to behavioral deficits, HI injured animals show reductions in gray matter volume (e.g., cortex and hippocampus), along with increased ventricular volume in post-mortem histological analysis [18,19,20,21,22].

Given deficits in behavior in the P7 HI rodent model, coupled with observed reductions in gray matter volume, we were interested in studying therapeutic agents that might attenuate injury and improve outcomes in this model. Caffeine is a promising agent that has recently gained attention as having potential to help mitigate some of the long-term behavioral deficits and neuropathology evident in HI injured infants. Caffeine is a non-selective adenosine receptor (AR) antagonist clinically used to increase respiratory drive in order to wean premature infants off ventilation and provide treatment of apnea of prematurity. Extensive research has characterized the regional distribution and function of adenosine receptor sub-types in the rodent brain, though much of this research derives from adult models and evidence suggests that AR function may differ in the neonatal brain [23]. The major adenosine receptor subtypes include adenosine (A) 1 receptor (A1R), A2AR, A2BR and A3R [24,25]. By adulthood, A1R mRNA is widespread in the brain, and is found at its highest levels in the hippocampus, cerebellum, and cerebral cortex [25]. A2AR mRNA is highly enriched in the striatum, globus pallidus, and is also found in astrocytes, microglia, and blood vessels throughout the brain [25]. A3Rs are present in nerve terminals of cortical and hippocampal cells, astrocytes, and microglial cells [25]. In the adult rodent brain, caffeine shows a high affinity for the A1R, A2AR and A3R, and a low affinity for A2BR. Caffeine has also been found to inhibit phosphoidesterase, promote the release of calcium from intracellular stores, and interfere with GABA-A receptor function, although these effects are seen at caffeine doses well outside of therapeutic ranges and thus are not candidate mechanism for the current model [25,26]. More data is clearly needed to fully characterize caffeine/adenosine receptor interaction in the neonatal brain, but caffeine has nonetheless shown beneficial neuroprotective effects following neonatal HI injury. For example, in addition to improving respiration, caffeine has also been found to improve the rate of survival without disability in premature infants, as well as decrease the incidence of cerebral palsy and neurodevelopmental delay [27,28,29,30,31]. Preterm infants treated with caffeine for two hours (via intravenous injection) also showed increased cortical activity as measured by EEG [32]. Finally, when neonatal mice were reared in an hypoxic environment from P3 to P14 with a dam provided *ad libitum* caffeine in drinking water, treated pups showed increased myelination, more normally arranged axons, increased proportion of oligodendrocytes, and decreased ventricular volume as compared to hypoxic mice who were not exposed to caffeine [33]. 

To our knowledge no studies have directly investigated the therapeutic benefits of caffeine on behavioral outcomes following P7 HI injury in the rat. Although other neuroprotective methods such as whole body or head cooling have been used to treat HI injury in infants, the development of a more widely available (*i.e.*, injectable) intervention might help improve infant outcomes following HI injury. Thus, the current study sought to investigate the effectiveness of caffeine treatment following HI injury in P7 rat pups. We hypothesized that rat pups treated with caffeine would show improved scores on the MWM and attenuated grey matter loss when compared to vehicle treated HI animals. 

## 2. Experimental Procedures

### 2.1. Subjects

Subjects were male Wistar rats born to time mated dams (Charles River Laboratories, Wilmington, MA, USA). Dams were shipped to the University of Connecticut on embryonic day 4 (E4), and were housed in tubs in an approved animal facility. Upon birth (P1), litters were culled to 10 pups (8 males and 2 females per litter). Only male pups were used in the current study, based on established sex differences between males and females following HI injury (with males showing a more robust pattern of deficits than females) [18]. Two female pups were retained in each liter to maintain normal maternal behavior. Subjects were weaned on P21 and pair housed with like-treated littermates until P55, when they were single housed for the duration of the study. All subjects were housed in a 12-h light/dark cycle with food and water available *ad* *libitum*. 

### 2.2. Induction of Hypoxia Ischemia

On P7, each pup was randomly assigned one of four treatment groups: HI vehicle (sterile saline), HI caffeine, sham vehicle, or sham caffeine. All treatment conditions were balanced across litters. For the HI procedure, pups were anesthetized with isoflurane (2.5%) and a midline incision was made longitudinally on the neck on the ventral side. The right common carotid artery was located and separated from surrounding tissue, cauterized, and the incision was sutured. Pups were marked with a footpad injection to indicate treatment group at weaning. Pups were then allowed to recover from the anesthesia under a warming lamp, and were returned to their dam for two hours to feed. Animals who were assigned to the sham condition were placed under anesthesia, but only had a midline incision with no manipulation of the carotid artery. Sham treated pups were allowed to recover and were also returned to the dam. Two hours after all pups had received the surgery, HI subjects were placed in an airtight oxygen container flowing 8% humidified oxygen (balanced with nitrogen) for a period of 120 min. Sham animals were placed in an open container with room air exposure for the same duration. 

### 2.3. Caffeine Administration

Immediately following the 120 min chamber exposure, animals received an intraperitoneal (i.p.) injection of either caffeine (10 mg/kg) or vehicle (sterile saline in comparable volume), and all pups were then returned to their dam. On P21, subjects were weaned and paired housed with like-treated littermates for later behavioral testing

### 2.4. Behavioral Testing

#### 2.4.1. Water Escape (P87)

A one-day water escape task was performed to assess for general motor deficits that might confound results. The water escape task involved the use of an oval tub (40.5 inches × 21.5 inches) filled with room temperature water and a visible platform at one end of the tub. Subjects were placed at the end of the tub opposite to the visible platform and were timed until they swam to and climbed on top of the platform. Latency to escape was recorded for each animal.

#### 2.4.2. Morris Water Maze (MWM; P90–P95)

MWM testing lasted for five consecutive days, and was performed in a 48 inch diameter hard plastic tub, with a 6 inch diameter platform submerged below the water line (so that is was not visible to the subjects). For each trial, the escape platform was located in the same quadrant (southeast) in the tub. Water was maintained at room temperature. The tub was surrounded by various extra-maze cues in the room (painted shapes on the walls, the experimenter, lights), and there were no intra-maze cues. Each testing day consisted of four trials in which the start position (north, south, east, west) varied. The start position never repeated in one day and the order varied randomly between testing days. Animals were given 45 s to locate and summit the submerged platform, and the latency to reach the platform was recorded for each subject for each trial. If an animal failed to reach the platform in 45 s, it was gently guided to the platform and allowed to sit for 5 s before being removed from the maze.

### 2.5. Histology

Upon the completion of behavioral testing, animals were anesthetized with Ketamine (100 mg/kg) and Xylazine (15 mg/kg) and transcardially perfused with 0.9% saline and 10% buffered formalin phosphate. Brains were removed and placed in the 10% buffered formalin phosphate. Prior to slicing, brains were placed in a 30% sucrose solution for cryoprotection for 24 h, and were then sliced in a coronal plane at 60 μm on a cryostat. Every third section was mounted on a slide and stained using cresyl violet. With the use of a Zeiss Imagine A.2 microscope and Micro Bright Field (MBF) Stereo Investigator computer software (Willinston, VT, USA) cortical volume was measured and calculated using Cavalier’s estimator. Due to complications with the slicing and/or staining procedures, some tissue sections were lost, so that there were not enough sections to create an accurate representation of total cortical volume in nine rat brains (5 HI saline and 4 HI caffeine). All measurements were performed blind to treatment group. 

### 2.6. Statistical Analysis

For all measures we employed planned paired comparisons among HI saline, HI caffeine, sham saline, and sham caffeine groups. Specific comparisons based on *a priori* hypotheses were made between: HI saline *vs.* sham (to confirm replication of HI effects); HI saline *vs.* HI caffeine (to assess any beneficial effects of caffeine); and HI caffeine *vs.* sham (to determine whether caffeine-treated subjects were equivalent to shams). In addition, we compared caffeine versus saline treated shams to ascertain any effects of caffeine on normal subjects, and/or to pool shams. 

Repeated Measure Multiple Analysis of Variance (ANOVA) was used to analyze MWM scores. Unless stated otherwise, all analyses were two-tailed. In comparing the HI saline to sham groups on behavioral and volumetric measurement outcomes, our lab has previously shown significant deleterious effects, and thus one-tailed analyses were used for these specific comparisons [19,20,21]. Variables analyzed and presented in the results section for the MWM task include Treatment (3 levels; sham [pooled sham saline and sham caffeine], HI saline, and HI caffeine) and Day (5 levels). For the MWM task, total latency (in seconds) to reach the platform for the four trials each day was the dependant variable. A “Learning Index (LI)” score was calculated to assess possible learning differences between groups (*i.e.*, an index of slope for each animal). The equation LI = (Day1 latency - Day2) + (Day 2 - Day 3) + (Day 3 - Day 4) + (Day 4 - Day 5) was applied to each animal, and scores were analyzed using a one-way ANOVA. Finally, a non-parametric Kruskal-Wallis ANOVA was used to analyze right cortical volumes (*i.e.*, the side of injury) by Treatment, using the same paired comparisons delineated above. SPSS 15.0 with a criterion of alpha 0.05 was used for all analyses. 

## 3. Results

In preliminary analysis we found no significant difference between sham saline and sham caffeine groups for MWM latencies [*F*(1,6) = 0.673, *p* > 0.05], and thus for further analysis these animals were pooled as “shams”.

### 3.1. Water Escape (P87)

A Univariate ANOVA revealed no significant effect of Treatment groups [*F*(2,35) = 0.87, *p* > 0.05] on water escape latencies indicating comparability in each group’s ability to see the platform, and to swim to it. 

### 3.2. Morris Water Maze (P90–P95)

An initial 5 (Day) x 3 (Treatment) repeated measures ANOVA revealed a significant between groups effect of Treatment for the three groups: HI saline, HI caffeine and sham [*F*(2,33) = 4.697, *p* < 0.05]. There was also a significant within subjects effect of Day [*F*(4,144) = 6.445, *p* < 0.01], reflecting decreasing latencies with learning. Additional ANOVAs were used to compare specific paired groups. We did not see a Treatment x Day interaction. 

**Figure 1:**
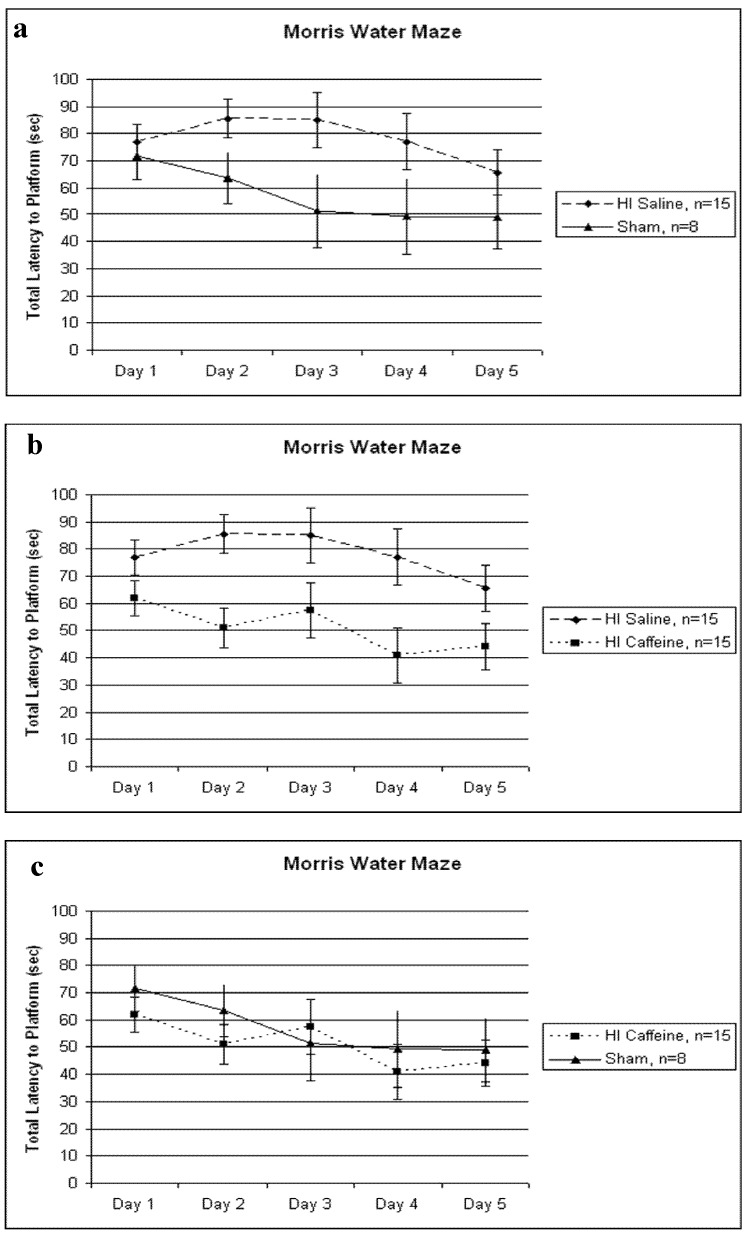
(**a**–**c**) Total latencies (in seconds) across 4 trials/day are shown. (**a**) A significant difference between HI saline and sham was seen [*F*(1,21) = 3.274, *p* < 0.05, one tailed], with HI saline performing significantly worse than shams. (**b**) A significant difference between HI saline and HI caffeine treated animals was seen [*F*(1,28) = 8.477, *p* < 0.01], with HI saline animals performing significantly worse than HI caffeine. (**c**) No significant differences in treatment were seen between HI caffeine and sham animals [*F*(1,21) = 0.363, *p* > 0.05]. For all graphs, error bars represent standard error.

A 5 (Day) x 2 (Treatment) repeated measures ANOVA revealed a significant Treatment difference between HI saline and sham treated animals [*F*(1,21) = 3.274, *p* < 0.05, one-tailed], with the HI saline group taking longer to reach the platform than shams (Figure 1a). A further 5 (Day) x 2 (Treatment) repeated measures ANOVA also revealed a significant Treatment difference between HI saline and HI caffeine treated animals [*F*(1,28) = 8.477, *p* < 0.01], with the HI saline group taking longer to reach the platform compared to the HI caffeine group (Figure 1b). Finally a 5 (Day) x 2 (Treatment) repeated measures ANOVA revealed *no* significant differences between HI caffeine and sham animals [*F*(1,21) = 0.363, *p* > 0.05] (Figure 1c). The within variable Day showed a trend for the HI saline and sham comparison [*F*(4,84) = 1.599, *p* = 0.09, one tailed], but was significant for the HI saline *vs.* HI caffeine comparison [*F*(4,112) = 2.7, *p* < 0.05] as well as for the HI caffeine and sham comparison [*F*(4,84) = 2.824, *p* < 0.05]. As above, these Day effects reflect learning.

Finally, to assess putative learning effects, a “Learning Index” was calculated for each animal. The results of a one-way ANOVA revealed no significant difference between groups on this measure [*F*(2,35) = 0.083, *p* > 0.05], however, scores were in the expected direction (shams > HI saline). 

In addition we performed a paired samples t-test between Day 1 versus Day 5 within each Treatment group. For the HI saline group this effect was non-significant [*t*(14) = 1.22, *p* > 0.05, one tailed], indicating there was no learning effect over Days. In the HI caffeine group, a paired samples *t*-test revealed a significant difference between Day 1 and Day 5 [*t*(14) = 1.88, *p* < 0.05, one-tailed]. Additionally, in the sham group, a paired samples *t*-test revealed a significant difference for learning as well, [*t*(7)=1.513, *p* < 0.05, one tailed]. 

### 3.3. Histology

#### Volumetric Analysis

Although the results of a Kruskal Wallis ANOVA did not yield any significant results for right cortical volume analysis, scores were in the expected direction (HI saline animals showing the smallest right cortical volume and sham animals showing the largest). It should be noted that a lower *n* was used for analysis in both the HI saline (*n* = 11) and HI caffeine (*n* = 10) groups, due to the fact that brain tissue showing severe injury or a high degree of ventriculomegaly became increasingly difficult to process. These technical confounds weakened the power of this analysis although again, the expected differences in means (HI saline < sham) were seen [for HI saline, M = 68.2 mm^3^ SD = 9.2 and for sham, M = 75.2 mm^3^ SD = 5.9].

## 4. Discussion

The current study provides evidence for protective effects of caffeine following a P7 HI injury in a rodent model. Outcomes include behavioral measures on the MWM and histological measures of right cortical volume. HI saline animals had significantly longer latencies to reach the platform on the MWM as compared to shams, replicating prior data [12,13,14,15,16,17]. Caffeine treatment immediately following HI injury preserved spatial memory function, with HI caffeine animals showing significantly shorter latencies to reach the platform than HI saline. Additionally, Learning Index scores were calculated for each animal across days, and analysis revealed no differences between the three groups. This suggests all animals could learn the task, but that HI saline animals performed worse overall. 

### 4.1. Adenosine in Neonatal HI

The cognitive deficits as well as neuropathological changes evident following neonatal HI injury are thought to be mediated in part by elevated adenosine levels following HI. Typically in neonatal rodents, interstitial adenosine levels (basal) are around 50 nM, but are believed to increase to upwards of 1000 nM during ischemic events [24,34]. Additionally, adenosine deaminase (ADA), a key enzyme to deaminate adenosine and thus maintain levels of adenosine, is also increased following neonatal HI in the cortex [35] and hippocampus [36]. This dramatic increase in adenosine may promote accelerated expression of apoptotic activity, leading to a rise in free radical and caspase formation [24]. During typical development in rodents, adenosine A1R increases in density between P9 and P15. Moreover, although receptors are found throughout the brain, highest levels are specifically seen in the cortex and hippocampus by adulthood [23]. Studies have shown that rodents treated with an A1 receptor agonist from P9 to P14 exhibit increased ventricular volume, reduced periventricular white matter volume, and a reduced number of neurons in the cortex and hippocampus [37] as well as decreased expression of myelin [24]. Theses changes are thought to reflect the fact that excess adenosine binding to the A1 receptor leads to the activation of hypoxia-inducing factor (HIF)-α, which in turn promotes the apoptotic cascade leading to cell death [24,38]. Additionally, adenosine accumulation following hypoxia is thought to be responsible for electrical suppression in the brain. For example, the release of adenosine was directly related to the depression of excitatory synaptic transmissions in hippocampal slices of P10–24 rats [39,40] and increased adenosine levels following hypoxia as measured *in vivo* in adult animals led to suppressed electrically evoked synaptic transmission [41]. Again these latter deleterious effects are thought to be mediated via activation of A1Rs. Consistent with the negative effects of A1 receptor activation in the neonatal brain, blockade of the A1 receptor has been found to exert protective effects in HI injured mice [42]. For example, knock-out mice deficient in the A1 receptor and reared in a hypoxic chamber from P3 to P14 showed decreased ventricular volume and increased white matter density as compared to wild-type HI mice that did express the A1 receptor [39]. 

Although the effects of adenosine expression and resulting brain damage following early HI injury appears to be primarily mediated through the A1 receptor, activation of the A2A receptor has also been implicated. For example, knock-out mice for the A2A (A2A -/-) receptor that had HI injury induced on P7 were evaluated three weeks to three months later, and brain injury was found to be exacerbated in the A2A -/- animals as compared to wild types. Furthermore, A2A -/- animals also showed impairments on rota-rod and beam walking tasks [43]. Since A2A receptors are located on the capillaries, an over-activation resulting from adenosine increases after HI injury could contribute to intraventricular hemorrhage via rupture of these vessels (although this has not been empirically demonstrated) [24]. In sum, A2A receptor activation is not well understood in development but may play a role in HI pathology. 

### 4.2. Caffeine Treatment Following Neonatal HI

In the adult brain, the effects of caffeine are primarily mediated through antagonism of A1Rs and A2ARs [25,44], where antagonism of A1 affects transmitter release and neuronal firing, and antagonism of A2As seems to affect dopaminergic transmission [44]. Additionally, caffeine has been shown to attenuate long-term potentiation in hippocampal slices [45]. The neuroprotective effects of caffeine following neonatal HI, however, are primarily via antagonism of the A1 receptor [34], consistent with evidence that AR antagonism appears to be mediated differently in the adult brain as compared to the embryonic fetal and neonatal brain [29]. Importantly, in the neonatal brain, A1R activation offers no protection in ischemia-induced damage but instead is associated with brain injury [23]. Studies have shown deleterious effects of caffeine (not seen in adults) during normal fetal brain development, including a wide array of behavior, neurochemical, and electrophysiologic changes [46,47,48,49,50]. However, serum caffeine levels in caffeine treated premature infants were found to be correlated with a change in pro-inflammatory and anti-inflammatory cytokine levels in the peripheral blood, with a greater concentration of anti-inflammatory molecules correlated with higher levels of caffeine (between 10 and 20 μg/mL). Additionally, reductions in interleukin (IL)-6 and tumor necrosis factor (TNF)-α (both pro-inflammatory) and increases in IL-10 (anti-inflammatory) were seen in these subjects [51]. Finally, activation of A2ARs and A2BRs (located on capillaries [25]) could induce capillary leakage, which might contribute to hemorrhages and HI. Thus antagonism of A2ARs and A2BRs may explain in part why caffeine treated infants show lower incidence of intra-ventricular hemorrhage (IVH) [24]. Caffeine treatment also down-regulates TNF-α and the release of cytokines in response to lipopolysaccharides (an endotoxin that elicits a large inflammatory response from the immune system; LPS) in term injured infants [52]. There are likely to be additional cellular factors involved in cognitive protection based on the work of others. For example, in cultured developing cortical neurons, caffeine enhanced CREB dependant gene expression and mediated the activity-dependant BDNF and TrkB expression [53]. Neonatal mice reared in hypoxia treated with caffeine also showed more normally arranged axon orientation and increased the proportion of immature oligodendrocytes [33]. Premature baboons treated with caffeine also showed improved myelination [34]. Similarly, a study investigating the effects of induced early life convulsions in P7 rodents, injured animals showed deficits in selective memory in adulthood and a loss of presynaptic glutamate terminals. Caffeine prevented these memory deficits and prevented the loss of nerve terminal markers in the hippocampus [54]. Although loss of gross brain volume is a significant complication following neonatal HI injury, it could be that the protective effects of caffeine allow in part for cellular reorganization and plasticity effects contributing to the rescue of behaviors. Additional studies are needed to confirm this hypothesis and to characterize how different cell types might reorganize themselves in order to prevent brain injury and resulting behavioral deficits following early HI injury. 

### 4.3. Central *versus* Peripheral Effects of Caffeine Treatment

As stated above, caffeine was originally used in premature infants to improve respiratory drive and treat apnea of prematurity by decreasing the threshold for sensitivity of hypercapnia, and increasing contractility of the diaphragm [55]. Caffeine has also been shown to lead to a decreased rate of bronchopulmonary dysplasia in treated compared to non-treated premature children [27] as well as improved cognition at 18 months. Due to improved and more consistent adequate blood oxygenation in these treated infants, it is not possible to conclude definitely that the neuroprotective effects of caffeine in this population are mediated exclusively by central brain related changes versus the elimination of intermittent hypoxia. Animal models expressing the A1 receptor show suppression of respiratory drive with adenosine, and stimulation of respiratory drive with caffeine, while in A1R knock-out mice for A1Rs, adenosine showed no suppressive effects on respiratory drive, and no stimulatory effects with caffeine [24]. Blockade of A2A receptors are also implicated in the neuroprotective role of caffeine. Specifically, in the medulla of the brainstem (an area implicated in cardiac function and respiratory drive), about half of the GABAergic neurons (measured by *in situ* hybridization and retrograde tracing) express mRNA for A2A receptors in the developing mouse brain. This suggests that A2A receptors might play an important role in respiration early in life as well [56]. While brain injury in premature infants are typically associated with repetitive respiratory-induced mild to moderate drops in brain oxygenation over a period of weeks, hypoxia brain injury in term infant are generally perinatal acute events, much like the acute injury used in our rodent model. Thus we hypothesize the neuroprotective effects of caffeine in our model are due to the direct effect of caffeine via the interaction with the adenosine receptor and not due to changes in breathing and long term improved oxygenation. 

## 5. Conclusions

Here, we replicated prior reports that HI injury in a P7 rodent leads to behavioral deficits on the MWM task, and provide new evidence that these deficits can be ameliorated with the use of caffeine treatment immediately following the induction of injury. Evidence suggests that these effects may be largely mediated via caffeine antagonism of A1 receptors, although this hypothesis must be empirically tested. In closing, additional studies are needed to determine the possible neuroprotective role of caffeine for other behavioral modalities, and neuroanatomical alterations, in both rodent models and human trials. Future studies could investigate the effects of delayed and/or multiple injections of caffeine, varied caffeine dosage, caffeine coupled with other therapeutic agents, or in combination with hypothermia treatment. 
